# Small bowel duplication cyst in the pediatric population—when to operate?

**DOI:** 10.1007/s00383-024-05959-8

**Published:** 2025-01-03

**Authors:** Yael Dreznik, Anastasia Almog, Maya Paran, Osnat Konen, Dragan Kravarusic

**Affiliations:** 1 Department of Pediatric and Adolescent Surgery, Schneider Children’s Medical Center of Israel, 14 Kaplan St, Petah Tiqwa, Israel; 2 Department of Radiology, Schneider Children’s Medical Center of Israel, Petah-Tiqwa, Israel; 3 Faculty of Medicine, Tel Aviv University, Tel Aviv, Israel

**Keywords:** Duplication cyst, Pediatric, Small bowel, Prenatal diagnosis

## Abstract

**Introduction:**

**Aim:**

The aim of the study is to determine the optimal timing for surgery in patients with small bowel duplications.

**Methods:**

A retrospective cohort study, including all patients younger than 18 years who were diagnosed with small bowel duplications from 2013 until 2024 in a single tertiary medical center, was performed. Patients’ demographics, duplication size and location, pathological results, and clinical outcomes were collected.

**Results:**

Sixteen patients (nine boys, seven girls) underwent laparoscopic-assisted resection of small bowel duplication at an average age of 3 years. A prenatal diagnosis was made in 11 patients, 10 (91%) of whom underwent elective surgery at a median age of 1.3 years. Overall, six patients required semi-elective or urgent surgery due to bowel obstruction, abdominal discomfort, or symptomatic anemia, with most (83%) lacking prenatal evaluation. Elective surgery patients had significantly smaller duplications (13 cm^3^ vs. 135 cm^3^). Post-operative recovery was satisfactory in all patients, with an average hospital stay of 6 days.

**Conclusion:**

In conclusion, asymptomatic, small duplication cysts in the small bowel of pediatric patients can be managed expectantly and can be operated after the first year of age. This approach is safe and allows for laparoscopic exploration in older infants, yielding satisfactory outcomes.

## Introduction

Small bowel duplication is a rare congenital anomaly that occurs in approximately 1 in 4500 live births. These duplications can be cystic or tubular and are most commonly found in the ileum, followed by the jejunum. They share a common blood supply with the adjacent bowel and are characterized by a muscular wall and epithelial lining resembling some portion of the gastrointestinal tract [1, 2].

Prenatal diagnosis of small bowel duplication is possible through routine ultrasonography, typically in the second or third trimester. The characteristic appearance is a cystic mass adjacent to the bowel. Fetal MRI may be used for further evaluation and to rule out other abdominal cysts.

Management of small bowel duplications generally involves surgical resection. However, there is ongoing debate regarding the optimal timing of surgery for asymptomatic cases detected prenatally or incidentally [3].

While some advocate for early intervention—sometimes immediately after birth—to prevent potential complications such as obstruction or bleeding [4, 5], there are still no clear recommendations regarding the timing of surgery of small bowel asymptomatic duplication cysts, that are diagnosed prenatally. Currently, the decision is often made on a case-by-case basis, considering factors such as the size, location, and potential for complications.

The aim of the study is to determine the optimal timing for surgery in patients with small bowel duplications based on our experience in the diagnosis, treatment, and follow-up of these patients.

## Methods

### Study design

A retrospective cohort study of all pediatric patients who underwent surgical resection of enteric duplication cyst between 2013 and 2024 in a tertiary center was conducted. The study included all patients who were diagnosed with small bowel duplication cyst. The data collected in this study included age at diagnosis, gender, duplication’s location, imaging findings, surgical details, post-operative complications.

### Data acquisition and analysis

The study was approved by the local institutional review board committee.

All data were collected via the computerized hospitalization records for each participating patient. Data including age, gender, clinical presentation, and imaging findings were collected. Operative data, including pathologic reports, and post-operative outcomes, were also collected.

All specimens were analyzed and processed by the Department of Pathology in our institute.

### Surgical technique

In all patients, surgery was performed using a laparoscopic approach, during which the small intestine was examined to locate the duplication. Subsequently, the umbilical incision was extended, the duplication was exteriorized, and resection of the duplication together with the involved intestinal segment was performed. An extracorporeal end-to-end anastomosis was then performed.

### Statistical analysis

Descriptive statistical methods were employed for the analysis of all collected data, including average, range, distribution, minimum, and maximum. A Shapiro–Wilk test was performed for continuous variables, to determine normal distribution. Accordingly, continuous variables were compared using the independent sample *t* test or Mann–Whitney test, for normally distributed and non-normally distributed variables, respectively. Pearson X^2^ test of independence or Fisher’s exact test was performed for categorical variables, as appropriate. Statistical significance was considered as a two-tailed *p* value of 0.05 or less. All the analyses were performed using Python.

## Results

### Patient and duplication cyst’s characteristics

Between the years 2013–2024, a total of 16 patients were diagnosed with small bowel duplication at our institution.

The study population was divided into patients for whom the diagnosis of duplication was made prenatally (prenatal diagnosis, “elective group”) and patients for whom the diagnosis was made due to abdominal symptoms, without prior known prenatal diagnosis (“non-elective group”).

In patients diagnosed prenatally, the patient was referred to a surgical clinic after birth for a follow-up ultrasound and subsequent follow-up including follow-up ultrasound at intervals of several months, provided the patient was asymptomatic. Surgery for these patients was scheduled electively after the age of 1 year on average, at 1.5 years old. Only one patient who was diagnosed prenatally was referred to semi-elective surgery at the age of 3 months due to intussusception—this patient had a duplication cyst that was located very close to the ileocecal valve. This patient was assigned, retrospectively, to the “non elective” group.

In the elective group, meticulous documentation was performed during clinic visits every few months (typically every 3 months), which included a clinical evaluation and a complete physical examination. In addition, symptoms potentially associated with the presence of the duplication—such as vomiting, restlessness, weight loss, signs of anemia, etc.—were ruled out.

In patients without prior known prenatal diagnosis, that were diagnosed postnatally due to complaints of abdominal pain, signs of bowel obstruction, or anemia (“non elective group”), surgery was performed shortly after the diagnosis.

Table 1 illustrates the key epidemiological findings of patients who were admitted to our institution with small bowel duplication.

**Table 1:** Patients and tumor characteristics

Characterization	Elective	Non-elective	*p* value
	*n* = 10	*n* = 6	
Median age, years (range)	1.3 (1–2.8)	2.58 (3 months, 18 years)	0.11
Gender (M,F)	5:5	4:2	0.63
Clinical data prior medical consultation			
Prenatal diagnosis	10/10	1/6	0.0014
Clinical complaints			
Abdominal discomfort	0/10	3/6	na
Anemia	0/10	1/6	na
Small bowel obstruction	0/10	2/6	na

*na* Not applicable

### Pre-operative radiological findings and clinical findings

All patients—in both groups—underwent ultrasound imaging prior to surgery. In three (3) of the patients, MRI was recommended in addition to the ultrasound to better characterize the duplication itself and its relationship to the intestine. In one patient, a CT scan was performed.

Imaging data are described in Table 2.

**Table 2:** Pre-operative radiological findings

Characterization	Elective	Non elective	*p* value
	*n* = 10	*n* = 6	
No. of patients that underwent radiological evaluation prior to surgery			
Prenatal ultrasound	10	1	na
Prenatal MRI	0	1	na
Pre-surgery ultrasound	10	6	na
Pre-surgery MRI	2	0	na
Pre-surgery CT	0	1	na
Average (mean) preoperative radiological measurements of the mass (range, cm^3^)	12.93 cm^3^ (1.5 cm^3^ – 43.38 cm^3^)	134.7 cm^3^ (12.5 cm^3^ – 330 cm^3^)	0.0232

*na* Not applicable

### Operative and post-operative data

The surgery was performed using a laparoscopic-assisted approach as outlined in the “Methods” section, with the objective of complete duplication resection and bowel anastomosis. The majority of surgeries were completed without post-operative complications, with the exception of one patient, from the elective group, who developed a fever without signs of surgical site infection or other source. All patients resumed oral intake on average 3 days post-surgery and were hospitalized for an average of 6 days.

In ten patients, the pathology reveled duplication cyst without ectopic mucosa. In six patients, a duplication cyst with ectopic gastric tissue was found, with no statistical significance within the groups (elective vs. non-elective).

All patients underwent a post-operative follow-up visits, which included physical examinations.

Data regarding operative and post-operative assessment are presented in Table 3.

**Table 3:** Pre-operative and post-operative assessment

Characterization	Elective	Non-elective	*p* value
	*n* = 10	*n* = 6	
Location of the duplication			
Jejunum (jejuno-jejunal anastomosis)	1	0	1.00
Ileum (ileo-ileal anastomosis)	3	4	0.302
Terminal ileum (ileo-colic anastomosis)	6	2	0.608
Length of stay (days, average)	6.5	6.1	0.108
Post-operative complications	1/10	0/6	1.00

All patients had at least one post-operative visit in the outpatient clinic 1 month following surgery. No referrals were documented in the study population regarding late complications, such as bowel obstruction.

## Discussion

Enteric duplication is a rare congenital anomaly. Its clinical presentation varies based on the location and characteristics of the lesion, and necessitating surgical resection, most often using a laparoscopic-assisted approach [6]. While duplication cysts can appear anywhere along the gastrointestinal tract, they are most frequently found in the small intestine [7].

Prenatal diagnosis has become more common in recent decades with the advancement of imaging capabilities during pregnancy [8]. Consequently, alongside the increase in diagnostic capabilities, the question has arisen regarding the optimal timing for surgical intervention in small bowel duplications when the patient is asymptomatic. Pediatric surgeons are, thus, faced with the question of whether to refer the patient for surgery soon after birth, within the first few months of life, or if it is possible to wait and refer the patient for surgery after the first year of life, provided that there is regular sonographic and clinical follow-up. To date, the research literature has not addressed specific criteria dictating planned elective surgery.

Recently, a review by Morris et al. [9] have addressed an updated summary of enteric duplications, although without clear recommendations regarding timing of surgery. Another large series of ileocecal duplications by Yan et al. [10] have summarized specific findings regarding the surgical management of such duplications, without mentioning issues such as pre-operative surveillance and referral for elective surgery.

In our study, we review the experience accumulated over more than a decade in managing patients with small bowel duplication only, to homogeneously examine the study population (without including patients with duplications located in other locations along the gastrointestinal tract such as gastric, duodenal or colonic duplications).

Sixteen patients with small bowel duplications were included in out cohort. These patients have undergone laparoscopic-assisted resection of the duplication itself with performing bowel anastomosis. The population was divided into patients who underwent elective surgery following a prenatal diagnosis, and patients who underwent urgent or semi-elective surgery (during hospitalization) due to a diagnosis made postnatally due to abdominal symptoms (anemia, bowel obstruction, and signs of abdominal discomfort).

The location of the duplication in both groups was not significantly different, with most cases being in the ileum. All patients were discharged home after an average hospital stay of 6 days, without major complications.

The focus of our findings in this study is the post-natal management of patients who underwent elective surgery. In these patients, the size of the duplication, as measured by imaging and during surgery, was significantly smaller compared to patients who underwent non-elective surgery (13cm^3^ vs 135 cm^3^). In this group, surgery was performed at an average age of 1.5 years, following regular follow-up in the outpatient clinic, with documentation of physical examinations and repeated imaging studies.

All these patients, except one who was reassigned to the non-elective group and had semi-elective surgery due to intussusception, were asymptomatic and had an uneventful course until elective surgery took place.

Thus, regarding the dilemma in the above literature concerning the recommended age for elective surgery when a duplication is diagnosed, our study found that patients who underwent elective surgery were younger compared to those who underwent urgent or semi-elective surgery. This finding strengthens the decision to safely operate on patients with small, asymptomatic duplications after at least 1 year of age.

Our study has several limitations—for example, its retrospective nature. However, this study is observational in nature, summarizing our experience in the management and follow-up of pediatric patients with small bowel duplication cysts.

In summary, our study, which includes a homogeneous cohort, demonstrates that asymptomatic, small duplication cysts in the small bowel of pediatric patients can be managed expectantly and referred to elective surgery after the first year of age. Such management is considerably safe and allows for laparoscopic exploration in older infants, with satisfactory results.

## Data Availability

No datasets were generated or analysed during the current study.
